# SARS-CoV-2 Mediated Endothelial Dysfunction: The Potential Role of Chronic Oxidative Stress

**DOI:** 10.3389/fphys.2020.605908

**Published:** 2021-01-15

**Authors:** Ryan Chang, Abrar Mamun, Abishai Dominic, Nhat-Tu Le

**Affiliations:** ^1^College of Arts & Sciences, Washington University in St. Louis, St. Louis, MO, United States; ^2^Wiess School of Natural Sciences, Rice University, Houston, TX, United States; ^3^Department of Molecular and Cellular Medicine, College of Medicine, Texas A&M University, College Station, TX, United States; ^4^Department of Cardiovascular Sciences, Center for Cardiovascular Regeneration, Houston Methodist Research Institute, Houston, TX, United States

**Keywords:** SARS-CoV-2, Cardiovascular, EndMT, senescence, inflammation, oxidative stress, mitochondrial dysfunction, endothelial cells

## Abstract

Endothelial cells have emerged as key players in SARS-CoV-2 infection and COVID-19 inflammatory pathologies. Dysfunctional endothelial cells can promote chronic inflammation and disease processes like thrombosis, atherosclerosis, and lung injury. In endothelial cells, mitochondria regulate these inflammatory pathways via redox signaling, which is primarily achieved through mitochondrial reactive oxygen species (mtROS). Excess mtROS causes oxidative stress that can initiate and exacerbate senescence, a state that promotes inflammation and chronic endothelial dysfunction. Oxidative stress can also activate feedback loops that perpetuate mitochondrial dysfunction, mtROS overproduction, and inflammation. In this review, we provide an overview of phenotypes mediated by mtROS in endothelial cells – such as mitochondrial dysfunction, inflammation, and senescence – as well as how these chronic states may be initiated by SARS-CoV-2 infection of endothelial cells. We also propose that SARS-CoV-2 activates mtROS-mediated feedback loops that cause long-term changes in host redox status and endothelial function, promoting cardiovascular disease and lung injury after recovery from COVID-19. Finally, we discuss the implications of these proposed pathways on long-term vascular health and potential treatments to address these chronic conditions.

## Introduction

Coronavirus disease 2019 (COVID-19), caused by severe acute respiratory syndrome coronavirus 2 (SARS-CoV-2), has resulted in a global public health crisis. With over 65.4 million cases and 1.51 million deaths worldwide, SARS-CoV-2 has been far more devastating than other coronaviruses like SARS-CoV and MERS-CoV (Petersen et al., 2020). While SARS-CoV-2’s average basic reproductive rate (*R*_0_, defined as the average number of infections caused by one infected individual) is still being evaluated, most estimates place SARS-CoV-2’s *R*_0_ at around 2.5 or higher (Liu et al., 2020a; Petersen et al., 2020). This high transmissibility, coupled with SARS-CoV-2’s low fatality rate (found to be around 3.61% in an analysis of worldwide infections), has contributed to the virus’s rapid spread (Khafaie and Rahim, 2020; Petersen et al., 2020). Severe cases of COVID-19 have led to disease processes such as acute respiratory distress syndrome (ARDS), lung injury, thrombosis, and cardiovascular disease (CVD) (Klok et al., 2020; Teuwen et al., 2020; Yang et al., 2020). Notably, some patients have developed cardiovascular complications even after recovering from COVID-19. A study analyzing 100 recovered patients found cardiac involvement and myocardial inflammation in 78 and 60 individuals, respectively, while another study of recovering patients found that 75.4% of the cohort demonstrated reduced pulmonary function (Huang Y. et al., 2020; Puntmann et al., 2020). Notably, both studies analyzed patients within a wide range of ages and found these high rates of post-recovery conditions were independent of the patients’ preexisting conditions (Huang Y. et al., 2020; Puntmann et al., 2020). The presence of long-term disease processes associated with COVID-19 is particularly relevant, as a study found that 94% of patients in Hubei province recovered from COVID-19, indicating a high recovery rate potentially accompanied by chronic cardiovascular dysfunction (Hoseinpour Dehkordi et al., 2020). While the long-term consequences of SARS-CoV-2 infection and their molecular mechanisms remain relatively understudied, they are of paramount importance to public health due to the widespread nature of the virus (Carfi et al., 2020).

Endothelial cells (ECs), which mediate tissue inflammatory response and control solute and macromolecule transfer between the blood and tissue, are heavily involved in COVID-19 pathologies (Feletou, 2011; Varga et al., 2020). Immunohistochemical staining of SARS-CoV-2-infected ECs showed evidence of apoptosis, inflammation, and endothelial dysfunction, phenomena thought to result from the lethal COVID-19 inflammatory cytokine storm (Varga et al., 2020). Notably, the signaling pathways related to inflammation are highly regulated by mitochondria in ECs, thus suggesting a key role for the organelle in COVID-19 and its associated inflammatory pathologies (Tang et al., 2014; Guzik et al., 2020). EC mitochondria, while not major metabolic contributors, release mitochondrial reactive oxygen species (mtROS) that serve as signaling molecules in maintaining homeostasis and regulating inflammatory pathways (Culic et al., 1997; Quintero et al., 2006; Tang et al., 2014). Due to their free radicals and associated high affinity for electrons, mtROS are potent oxidizers that, at high levels, can directly damage DNA, proteins, and lipids or activate downstream pathways that promote inflammation and endothelial dysfunction (Li et al., 2013). Excess mtROS can also induce premature aging through oxidative telomere damage and promote the senescence-associated secretory phenotype (SASP), a state characterized by chronic inflammation and increased risk for cardiovascular disease and ARDS (Li et al., 2013; Powter et al., 2015; Chen and Dugas, 2019; Morty and Prakash, 2019). Due to less efficient transfer of electrons, dysfunctional mitochondria produce excessive levels of mtROS (Li et al., 2013). Mitochondrial dysfunction in ECs has thus emerged as a novel and important area of study in SARS-CoV-2 pathogenesis (Saleh et al., 2020).

**FIGURE 1:**
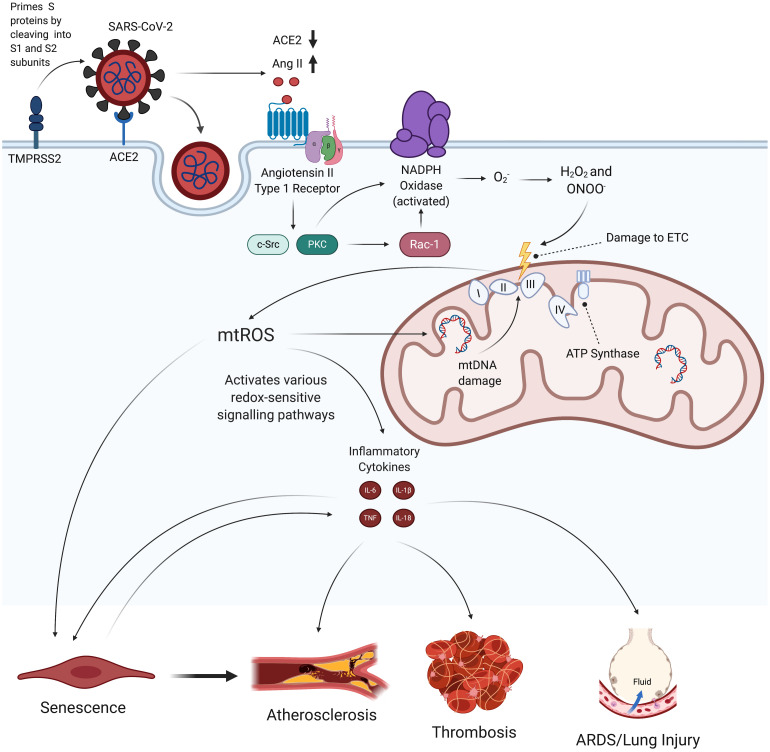
Hypothesized effects of SARS-CoV-2 on mitochondrial dysfunction, increased mtROS production, and subsequent endothelial pathologies. SARS-CoV-2 infection begins when its spike proteins are proteolytically primed by TMPRSS2, which enables them to bind to ACE2 and initiates viral endocytosis into the EC. This increases the amount of Ang II binding to AT_1_R, which, in turn, activates NADPH oxidase and subsequently induces increased production of mtROS via oxidative and nitrosative damage to the ETC. These excess mtROS mediate signaling pathways that elevate production of inflammatory cytokines. mtROS also inflict mitochondrial and nuclear damage that perpetuate mitochondrial dysfunction and further promote inflammatory senescence. These self-feeding interactions greatly increase the risk of atherosclerosis, thrombosis, and lung injury in people infected by SARS-CoV-2. TMPRSS2, transmembrane protease, serine 2; ACE2, angiotensin-converting enzyme 2; Ang II, angiotensin II; AT_1_R, angiotensin type 1 receptor; ETC, electron transport chain; mtROS, mitochondrial reactive oxygen species; IL, interleukin; TNF, tissue necrosis factor; ARDS, acute respiratory distress syndrome.

**FIGURE 2:**
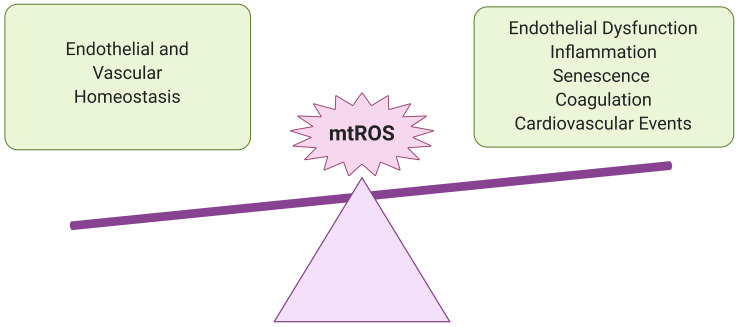
Importance of redox balance in ECs. Understanding the potential impact of SARS-CoV-2 infection on EC mtROS levels is crucial for predicting adverse cardiovascular and pulmonary events in recovered COVID-19 patients. At lower levels, mtROS are essential for maintaining endothelial homeostasis and regulating immune responses to infection and other environmental stresses. However, in excess, they can promote chronic inflammation and endothelial dysfunction, persistently increasing risk for vascular pathologies.

We propose that, by inducing mitochondrial dysfunction and oxidative stress, SARS-CoV-2 may initiate a feedback loop that promotes a chronic state of inflammation and endothelial damage even after viral particles have left the body. In this proposed mechanism, SARS-CoV-2 first induces activation of NADPH oxidase, which produces superoxide (O_2_^–^), a ROS that is involved in reactions that damage the electron transport chain (ETC) (Li et al., 2013; Nguyen Dinh Cat et al., 2013). The increased oxidative stress and inflammation resulting from this mitochondrial dysfunction then initiate a feedback loop that perpetuates NADPH oxidase activation, mitochondrial dysfunction, inflammatory cytokine production, SASP, and loss of EC identity (Figures 1, 2, and 6). This review discusses how this persistent state of oxidative stress and inflammation induced by SARS-CoV-2 infection increases risk for EC dysfunction, which would result in cardiovascular disease and respiratory failure. Given these hypothesized long-term consequences of SARS-CoV-2 infection on the vasculature, treating chronic oxidative stress and inflammation in ECs may be critical to preventing future health complications among the millions of people currently diagnosed with COVID-19 (Petersen et al., 2020).

## Sars-CoV-2 and the Endothelial Cells

### Role of ECs Under Physiological and Pathological Conditions

Under homeostatic conditions, ECs maintain vascular integrity and barrier function, while also controlling coagulation by producing an anti-coagulant glycocalyx layer and expressing anti-coagulant factors (Pober and Sessa, 2007; Teuwen et al., 2020). ECs mediate the inflammatory response to environmental stress factors like infection and injury by secreting inflammatory cytokines and recruiting immune cells, allowing for the exchange of fluid between the bloodstream and tissue (Krishnaswamy et al., 1999; Mai et al., 2013). Thus, ECs are key players in inflammatory pathologies, such as ARDS, thrombosis, and atherosclerosis (Zimmerman et al., 1999; Rajendran et al., 2013; Tang et al., 2014; Teuwen et al., 2020). One study found that SARS-CoV-2 infected ECs displayed higher levels of Von Willebrand Factor, an inflammatory marker that recruits leukocytes to ECs in response to inflammation (Kawecki et al., 2017; Helms et al., 2020). Autopsy of Mount Sinai Hospital patients confirmed that several deceased COVID-19 patients exhibited thrombosis, hyperinflammation, endothelial dysfunction, and diffuse alveolar damage, further supporting the role of inflammation and ECs in COVID-19 pathologies (Bryce et al., 2020).

### Initial SARS-CoV-2 Infection via Endothelial ACE2

Extensive research has been conducted into angiotensin-converting enzyme 2 (ACE2), which is highly expressed on the surface of ECs and serves as the primary entry mechanism for SARS-CoV-2 (Varga et al., 2020). The homeostatic role of ACE2 is to bind to angiotensin II (Ang II) and cleave it into angiotensin 1-7, a product that has been shown to exert protective effects on the heart and blood vessels by binding to G-protein coupled MAS1 receptor, which subsequently downregulates inflammatory signaling pathways (El-Hashim et al., 2012; Jiang et al., 2014b; Kai and Kai, 2020). Viral entry begins when TMPRSS2, a serine protease that is also highly expressed on the surface of ECs, primes coronavirus spike proteins via proteolytic cleavage (Hoffmann et al., 2020; Strope et al., 2020; Teuwen et al., 2020). SARS-CoV-2 spike proteins then bind to ACE2, which initiates endocytosis of the virus (Hoffmann et al., 2020). SARS-CoV-2 binding to ACE2 also downregulates ACE2 expression through a mechanism that, while still being explored, is hypothesized to result from spike protein activation of the metallopeptidase ADAM17, which cleaves the ACE2 ectodomain (Lambert et al., 2005; Miesbach, 2020; Wang K. et al., 2020; Xu et al., 2020). It has also been suggested that the interaction between ACE2 and viral glycoproteins leads to the creation of an ACE2 receptor-glycoprotein complex that results in ACE2 downregulation, although this proposal has yet to be fully explored (Glowacka et al., 2010). The downregulation of ACE2 is thought to increase levels of Ang II that are able to bind to the angiotensin II type 1 receptor (AT_1_R), since there are fewer ACE2 binding sites on the cell to degrade Ang II into the anti-inflammatory Ang 1-7 (Kuba et al., 2005; Escobales et al., 2019; Chung et al., 2020; Vaduganathan et al., 2020). Supporting this theory, a clinical study of 12 patients found that those infected with SARS-CoV-2 demonstrated higher plasma levels of Ang II compared to healthy controls. This phenomenon also correlated with increased viral load, reinforcing the idea that SARS-CoV-2 infection increases Ang II levels outside the cell that are able to bind to AT_1_R (Doughan et al., 2008; Liu et al., 2020b). As will be discussed in later sections, increased binding of Ang II to AT_1_R may be responsible for inducing EC oxidative stress, which, in turn, promotes inflammation, EC dysfunction, and increased risk for vascular disease processes (Figure 1; Lovren et al., 2008; Lee et al., 2019).

## Potential Role of Sars-CoV-2 in EC Mitochondrial Dysfunction

### mtROS Production in ECs

Given the focus on SARS-CoV-2’s effect on ECs, it is important to also examine the role of endothelial mitochondria in viral pathologies, as mitochondrial dysfunction has numerous negative implications for EC health (Chen and Dugas, 2019). In ECs, mitochondria are well-established signaling and metabolic organelles, and dysfunctional mitochondria have been linked to disease states like atherosclerosis and hypertension, making them key organelles for regulating EC health through production of mtROS (Tang et al., 2014). The main source of mtROS in ECs is electron leakage at ETC complexes I and III that reduces oxygen to O_2_^–^, some of which is later converted to hydrogen peroxide (H_2_O_2_) by superoxide dismutases (SODs) (Jastroch et al., 2010; Li et al., 2013). Another mechanism that fuels production of mtROS is the mitochondrial ATP-sensitive potassium channel (mitoK_ATP_), which allows influx of K^+^ into the organelle and subsequently increases the pH of the mitochondrial matrix (Andrukhiv et al., 2006; Garlid et al., 2013; Tang et al., 2014). This increased pH stabilizes the semiquinone radical, an intermediate in the one-electron transport necessary to reduce oxygen to O_2_^–^, thus facilitating increased production of mtROS (Selivanov et al., 2008). It has also been suggested that mitochondrial NADPH oxidase 4, which transfers electrons from NADPH to oxygen to form O_2_^–^, is another source of mtROS, although its localization to mitochondria in all ECs is not fully understood or confirmed (Block et al., 2009; Case et al., 2013; Tang et al., 2014).

### Mitochondrial Regulation of EC Health via mtROS

**FIGURE 3:**
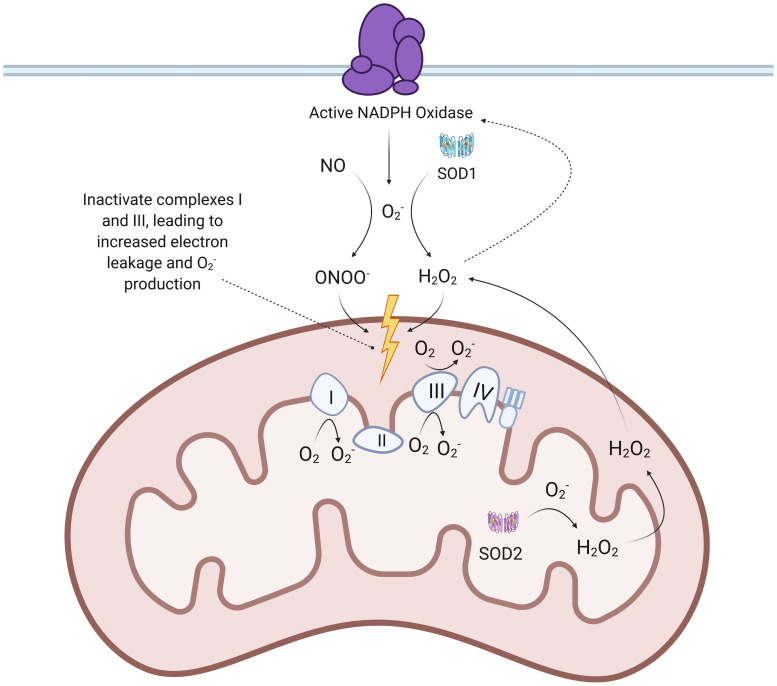
Proposed mechanisms of SARS-CoV-2 mediated increase in mtROS production. When SARS-CoV-2 spike proteins bind to the ACE2 receptor to enter ECs, ADAM17 is thought to be activated and cleave the ACE2 ectodomain, leading to downregulation of ACE2. This leads to an accumulation of Ang II that binds to AT1R, a G-protein-coupled receptor. The binding of Ang II to AT1R activates downstream effectors like c-Src, PKC, and Rac-1, which facilitate assembly and activation of NADPH oxidase. NADPH oxidase produces ${\text{O}}_{2}^{-}$ that reacts with NO to form ONOO^–^ and is also converted to H_2_O_2_ by SOD1. ONOO^–^ and H_2_O_2_ both inactivate ETC complexes, which results in increased production of ${\text{O}}_{2}^{-}$. Mitochondrial SOD2 can then convert ${\text{O}}_{2}^{-}$ to H_2_O_2_, which is able to diffuse across membranes. TMPRSS2: transmembrane protease, serine 2; ACE: angiotensin-converting enzyme; ETC: electron transport chain; mtROS: mitochondrial reactive oxygen species; SOD: superoxide dismutase; c-Src: proto-oncogene tyrosine-protein kinase Src; PKC: protein kinase C.

At low levels, mtROS regulate homeostatic processes like EC autophagy, immunity, and inflammation through oxidative signaling pathways (Tang et al., 2014). mtROS oxidatively activate Fyn and Ras proteins that phosphorylate the redox-sensitive p90 Ribosomal S6 Kinase (p90RSK), setting off a kinase cascade that leads to ERK5 S496 phosphorylation (Abe et al., 2000; Vu et al., 2018). Phosphorylated ERK5 then upregulates phosphorylative activation of NF-kB, leading to production of proinflammatory cytokines such as tissue necrosis factor (TNF) and interleukin 6 (IL-6) (Liu et al., 2017; Vu et al., 2018). mtROS can further promote inflammation by oxidizing thiols and inducing formation of a disulfide bond in the NLRP3 inflammasome, which, when fully assembled and activated, produces the IL-1β and IL-18 proinflammatory cytokines (Bae and Park, 2011; Zhou et al., 2011; Abais et al., 2015). These inflammatory cytokines increase expression of adhesion molecules that bind to leukocytes and recruit immune cells, such as macrophages, neutrophils, and T cells, to the site of inflammation (Mai et al., 2013; Ragab et al., 2020). mtROS also mediate cellular adaptation to hypoxia by preventing hydroxylation-associated inhibition of hypoxia-inducible factors, which translocate to the nucleus and increase transcription of genes involved in red blood cell production, angiogenesis, and glycolytic enzymes (Bell et al., 2007; Sena and Chandel, 2012; Schieber and Chandel, 2014). While these processes are essential for cellular responses to environmental stresses, it is also necessary for cells to remove excess mtROS, as elevated levels of mtROS can inflict severe oxidative damage on proteins, DNA, and lipids, which induces mitochondrial dysfunction and inflammation (Tang et al., 2014). The primary mechanism by which cells prevent the accumulation of mtROS involves rapidly inducible superoxide dismutases (SODs) that convert O_2_^–^ to H_2_O_2_, which is then reduced to H_2_O by antioxidants such as catalase and glutathione (Garlid et al., 2013; Tang et al., 2014). Inhibition and dysregulation of these antioxidant pathways lead to increased levels of ROS, resulting in oxidative stress (Figure 3).

### Mitochondrial Dysfunction in Endothelial Pathologies

Dysfunctional mitochondria, characterized by dysfunctional ETC activity and lower ATP production, are known to produce higher levels of mtROS (Davidson and Duchen, 2007; Murphy, 2013). This increased oxidative stress and subsequent upregulation of inflammatory cytokine production can facilitate endothelial dysfunction, which increases endothelial permeability and aggregation of pro-thrombotic platelets (Stenvinkel, 2001; Davignon and Ganz, 2004). Excessive recruitment of immune cells in response to increased inflammation is thought to be one of the primary mechanisms responsible for tissue damage and lung injury in the COVID-19 cytokine storm (Ragab et al., 2020; Ye et al., 2020). Furthermore, both ROS and inflammatory cytokines (TNF and IL-6) disrupt the endothelial barrier between blood vessels and tissue by activating the actin-myosin contractile apparatus, which pulls on the cadherin junctions that link adjacent ECs inward and away from each other (Millar et al., 2016). The endothelial barrier can be further weakened by EC apoptosis (Kluge et al., 2013; Chambers et al., 2018). One of the primary ways mtROS initiates EC apoptosis is by oxidizing intermembrane cardiolipin, which normally has a high affinity for the electron shuttle cytochrome *c* (Garrido et al., 2006). This causes cytochrome *c* to be released into the intermembrane space and cytosol, where it allosterically activates apoptosis-protease activating factor 1, which recruits and activates caspases that eventually execute apoptosis through mass proteolysis (Garrido et al., 2006; Bratton and Salvesen, 2010). This loss in endothelial barrier stability is considered the driving force behind the increased pulmonary edema observed in lung injury and ARDS (Millar et al., 2016; Fan et al., 2020). Thus, mitochondrial dysfunction and subsequent dysregulation of mtROS production represent a potential contributor to the lethal cytokine storm and inflammatory pathologies associated with SARS-CoV-2 infection, which will be discussed in greater detail in later sections.

### Proposed Mechanisms of SARS-CoV-2-Induced Mitochondrial Dysfunction

Given the extensive damage mtROS can cause, it is critical to examine how SARS-CoV-2 infection can induce mitochondrial dysfunction in ECs. The increase in Ang II levels may be one of the mechanisms responsible for elevating mtROS production and subsequent EC pathologies, as Ang II has been shown to increase ROS production via increased NADPH oxidase (Nox) activation (Liang et al., 2018). Past studies have found that a 4-h, 200 nmol/L treatment of Ang II increased mitochondrial H_2_O_2_ production by 1.5-2 times in ECs, an increase mediated through Nox-generated ROS (Doughan et al., 2008). Ang II binding to AT_1_R, which is coupled to a heterotrimeric G-protein, leads to increased production of diacyl glycerol that activates protein kinase C (Lipp and Reither, 2011). Protein kinase C regulates the phosphorylative activation and translocation of Nox subunit p47phox to the cell membrane (Choi et al., 2008; Wynne et al., 2009; Lipp and Reither, 2011; Nguyen Dinh Cat et al., 2013). Importantly, protein kinase C and c-Src can also activate the small GTPase Rac1 through downstream phosphorylation of GDP to GTP (Figure 1; Harrison et al., 2003; Ohtsu et al., 2006; Gianni et al., 2008). Rac1 then further promotes NADPH oxidase activity by binding to Nox activator p67phox, which contains an activation domain necessary for O_2_^–^ production via electron flow in a GTP-dependent manner (Ueyama et al., 2006; Welch, 2008; Nguyen Dinh Cat et al., 2013; Schroder et al., 2017). Research also indicates that increased levels of Ang II are associated with increased transcription and expression of Nox isoforms and subunits (Sachse and Wolf, 2007; Brown and Griendling, 2009; Nguyen Dinh Cat et al., 2013; Panday et al., 2015). Further research is required to understand the precise control and kinetics of Nox activation following AT_1_R activation.

O_2_^–^ created by the NADPH oxidase then react with NO to form ONOO^–^, which is known to damage and inactivate the mitochondria’s respiratory complexes through tyrosine nitration and cysteine oxidation (Pacher et al., 2007; Doughan et al., 2008; Dikalov and Nazarewicz, 2013). This inactivation of ETC complexes, one of the hallmark initiators of mitochondrial dysfunction, results in increased reduction of oxygen and subsequent mtROS production due to accumulation of electrons that are unable to be used for ATP production (Pacher et al., 2007; Doughan et al., 2008; Dikalov and Nazarewicz, 2013; Murphy, 2013; Korge et al., 2016). NO can also nitrate ETC complexes but does not contribute as much to increased production of mtROS due to its most sensitive target being complex IV, which does not synthesize O_2_^–^ (Tengan and Moraes, 2017). Furthermore, H_2_O_2_ formed by dismutated O_2_^–^ can permeate the mitochondrial membrane and oxidize the thiol side chain of cysteine, causing further ETC dysregulation and increased mtROS production (Pacher et al., 2007; Mailloux et al., 2014). Ang II and G-protein-mediated activation of protein kinase C epsilon (PKC-ε) has also been shown to result in phosphorylation and opening of mitoK_ATP_, thus further increasing mtROS production (Nunez et al., 2017).

### Dysfunctional Mitochondria and Excess mtROS Form Self-Feeding Loop That Pose Risk for Chronic EC Dysfunction

The induction of mitochondrial dysfunction poses chronic EC health risks beyond the initial response to viral infection. Notably, elevated levels of mtROS form a dangerous feedback loop with dysfunctional mitochondria, perpetuating a cycle that promotes inflammation in ECs (Zorov et al., 2014). The aforementioned formation of ONOO^–^ also occurs within the mitochondrial matrix through mitochondrial O_2_^–^, which further damages the ETC (Pacher et al., 2007). The activation of p90RSK by mtROS and subsequent ERK5 S496 phosphorylation may further exacerbate oxidative stress, as evidenced by increased levels of redox-sensitive p90RSK enzymatic activity, by inhibiting transcription of the antioxidant nuclear factor erythroid 2-related factor 2 (Singh et al., 2019). mtROS-mediated NLRP3 inflammasome activation and redox-sensitive kinase cascades produce inflammatory cytokines like IL-1β and TNF, which can activate NADPH oxidase and further perpetuate oxidative stress (Figure 1; Kaur et al., 2004; Yang et al., 2007). It has also been suggested that H_2_O_2_ can stimulate NADPH oxidase’s production of O_2_^–^, representing another potential feedback loop triggered by SARS-CoV-2 infection (Figure 3; Doughan et al., 2008). H_2_O_2_ also oxidizes thiols in the zinc finger of PKC-ε, which further increases mtROS production by opening mitoK_ATP_ and allowing for K^+^ influx (Garlid et al., 2013).

Furthermore, O_2_^–^ generated by both the mitochondria and NADPH oxidase can induce oxidative modifications to DNA sugar-phosphate backbones, resulting in mutations and DNA damage (Li et al., 2013; Dominic et al., 2020b). The damage to mitochondrial DNA (mtDNA) caused by ROS is thought to further induce mitochondrial dysfunction due to improper assembly of ETC complexes (Ide et al., 2001; Doughan et al., 2008). However, this is not conclusively known, as it has been reported that accumulation of mtDNA mutations in mice resulted in reduced ATP production but showed no evidence of increased ROS damage (Kujoth et al., 2005; Trifunovic et al., 2005). Notably, high amounts of mtDNA mutations are correlated with greater induction of apoptotic markers, suggesting that the persistent, feedback loop-fueled production of mtROS still poses a great risk for EC apoptosis and subsequent development of ARDS edema and lung injury (Kujoth et al., 2005).

H_2_O_2_, the mtROS that more easily diffuses across membranes, reacts with intracellular iron to produce highly reactive hydroxyl radicals that induce nuclear DNA strand breaks (Mello Filho et al., 1984; Balasubramanian et al., 1998; Lee et al., 2011). Zinc-dependent Poly ADP-ribose polymerase-1 (PARP-1) is one of the main DNA damage sensors that binds to these single- and double-strand breaks and activates various DNA repair mechanisms through ADP-ribosylation (Hocsak et al., 2017; Ray Chaudhuri and Nussenzweig, 2017; Dominic et al., 2020b). However, it can be over-activated when there is extensive DNA damage resulting from persistent oxidative stress (Dominic et al., 2020a). This increase in PARP-1 activation also increases expression of c-Jun N-terminal kinases (JNK) and p38 mitogen-activated protein kinase in response to cellular stress (Hocsak et al., 2017; Dominic et al., 2020a). Activated JNK phosphorylates c-Jun, which then helps form the AP-1 transcription factor that suppresses SOD gene expression, thus exacerbating the accumulation of O_2_^–^ (Riera et al., 2015). Activation of JNK and p38 mitogen-activated protein kinase pathways is also associated with changes in mitochondrial membrane potential (MMP) and apoptosis (Hocsak et al., 2017). Critically, extreme changes in MMP in either direction compromise the cell’s antioxidant system, which also increases mtROS production (Li et al., 2013). These wide-ranging causes and consequences of elevated mtROS levels demonstrate the importance of investigating potential SARS-CoV-2-activated feedback loops that exist between mtROS production, mitochondrial dysfunction, and inflammation (Figure 1).

### Dysfunctional Mitochondrial Metabolism

A phenomenon that has gone relatively understudied is SARS-CoV-2’s effect on mitochondrial metabolism, which may be relevant because many viruses increase glycolytic metabolism and TCA intermediate concentrations in ECs to ensure successful replication (Goodwin et al., 2015; Sanchez and Lagunoff, 2015). Interestingly, lipid metabolic analysis of SARS-CoV survivors found elevated levels of cysteine, aspartic acid, and alanine, which are all intermediates in the tricarboxylic acid (TCA) cycle (Wu et al., 2017). This study is of particular interest because it suggests that SARS-CoV-2, which has proteins that are highly homologous to those of SARS-CoV, may cause similar long-term metabolic changes that further promote chronic oxidative stress and inflammation in ECs (Xu et al., 2020). Increasing TCA cycle metabolism poses a risk for even greater endothelial dysfunction, as high levels of metabolites overburden the ETC, resulting in increased electron leakage and associated mtROS production (Groschner et al., 2012). Additionally, succinate oxidation by Complex II drives production of inflammatory IL-1β in macrophages, while an accumulation of fumarate binds to the antioxidant glutathione and mediates an increase in oxidative stress (Zheng et al., 2015; Martinez-Reyes and Chandel, 2020). Notably, while succinate levels were not found to be increased in the serum of COVID-19 patients, the authors of the study in question hypothesized that succinate levels may have been significantly lowered by the patients’ intense respiratory therapy at the time of blood draw (Thomas et al., 2020). Of further interest is the finding that lipopolysaccharide, which is known to increase succinate levels, has been correlated with more severe COVID-19 outcomes; for example, newer smokers and patients with pneumonia and other lung infections – demographics associated with high lipopolysaccharide levels – are known to be at greater risk of COVID-19 complications and mortalities (Petruk et al., 2020).

**FIGURE 4:**
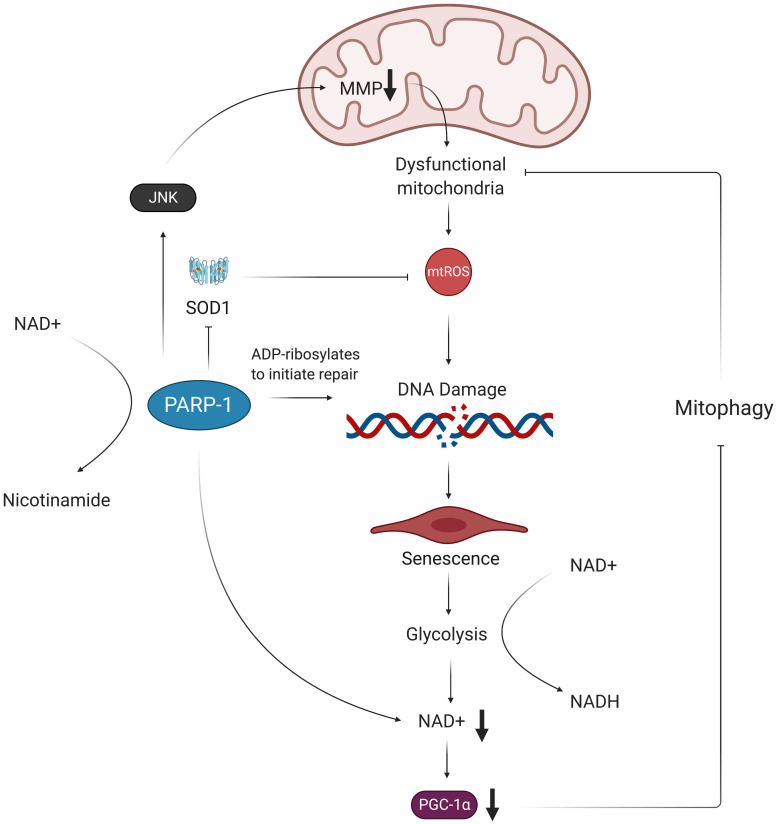
Oxidative stress results in NAD+ depletion and perpetuates mitochondrial dysfunction. Oxidative damage of telomeric DNA invokes the PARP-1 DNA damage response, which consumes NAD+ in order to ADP-ribosylate DNA and proteins. This activation of PARP-1 exacerbates accumulation of mtROS by decreasing expression of SOD1 and decreasing MMP. The excess mtROS also induce senescence via oxidative telomere damage, which leads to a metabolic shift toward glycolysis. This increased glycolytic activity results in further NAD+ depletion in ECs, which, in turn, leads to decreased expression of PGC-1α. Without PGC-1α to promote expression of mitophagy-related genes, the dysfunctional mitochondria are not properly removed from the cell and are thus able to continue producing more mtROS, contributing to a chronic state of oxidative stress in ECs. MMP, mitochondrial membrane potential; mtROS, mitochondrial reactive oxygen species; NAD+, nicotinamide adenine dinucleotide; NADH, nicotinamide adenine dinucleotide + hydrogen; SOD1, superoxide dismutase 1; PARP-1, poly ADP-ribose polymerase 1; JNK, c-Jun N-terminal kinase; PGC-1α, peroxisome proliferator-activated receptor gamma coactivator 1-alpha.

Furthermore, low NAD + /NADH ratios are associated with vascular dysfunction and have been observed in senescent ECs (Csiszar et al., 2019; Dominic et al., 2020b). This reduction in NAD + levels is compounded by the shift of infected and senescent cells to more glycolytic metabolism, as glycolysis takes up NAD + to yield NADH for electron transfer in mitochondrial ATP synthesis (Birch and Passos, 2017). These findings are corroborated by the fact that activated macrophages, which also shift toward glycolysis for ATP production, show increased levels of mtROS and inflammation (Mills et al., 2016). Low NAD + /NADH ratios are characteristic of minimal need for ATP and promote passing of electrons from complex I to oxygen, thus producing O_2_^–^ (Widlansky and Gutterman, 2011). The DNA damage repair initiated by PARP-1 further exacerbates the reduced amount of NAD + , as PARP-1 requires NAD + to donate ADP-ribose (Fang et al., 2014; Birch and Passos, 2017). This NAD + depletion leads to decreased expression of NAD + -dependent SIRT1, a deacetylase that enhances activity of the transcriptional coactivator PGC-1α (Gomes et al., 2013; Zhou et al., 2017; Dominic et al., 2020b). PGC-1α increases expression of genes involved in mitophagy, the process by which dysfunctional or injured mitochondria are destroyed by autophagosomes. Thus, PGC-1α reduction resulting from NAD + depletion may further perpetuate mitochondrial dysfunction in ECs following SARS-CoV-2 infection (Figure 4; Fang et al., 2014; Vainshtein et al., 2015).

These alterations in endothelial metabolism have been associated with vascular dysfunction and atherosclerosis in aging patients (Sabbatinelli et al., 2019). Thus, addressing the glycolysis pathway may be a crucial point of intervention, as indicated by the inhibition of the pro-inflammatory IL-1β in mice treated with the glycolytic inhibitor 2DG (Tannahill et al., 2013). However, there are some conflicting reports that suggest endothelial senescence has no effect on, or may even actually reduce, glycolytic activity in ECs, making the precise role of senescence on EC glycolytic metabolism controversial (Urata et al., 2019). Overall, current evidence suggests SARS-CoV-2 infection engenders dysregulated mitochondrial metabolism in ECs, thus contributing to the proposed pathways of infection-induced oxidative stress and mitochondrial and endothelial dysfunction (Table 1).

**TABLE 1:** Role of metabolites in oxidative stress and mitochondrial dysfunction.

Metabolites	Potential Changes in Senescent ECs	Effect on Oxidative Stress	References
NAD + /NADH	Lower NAD + /NADH ratios due to shift toward glycolysis	Decreased activation of PARP, which helps repair oxidative stress-induced DNA damage	Birch and Passos, 2017
Cysteine, aspartic acid, alanine	Increased levels due to higher TCA activity	More electron donors result in increased ROS production in the ETC	Groschner et al., 2012
Succinate	Increased Levels	Oxidized by Complex II, leads to production of inflammatory cytokines	Zheng et al., 2015; Martinez-Reyes and Chandel, 2020
Fumarate	Increased Levels	Binds to glutathione and mediates increase in oxidative stress	Zheng et al., 2015; Martinez-Reyes and Chandel, 2020

### Direct Viral Localization May Further Induce Mitochondrial Dysfunction

**FIGURE 5:**
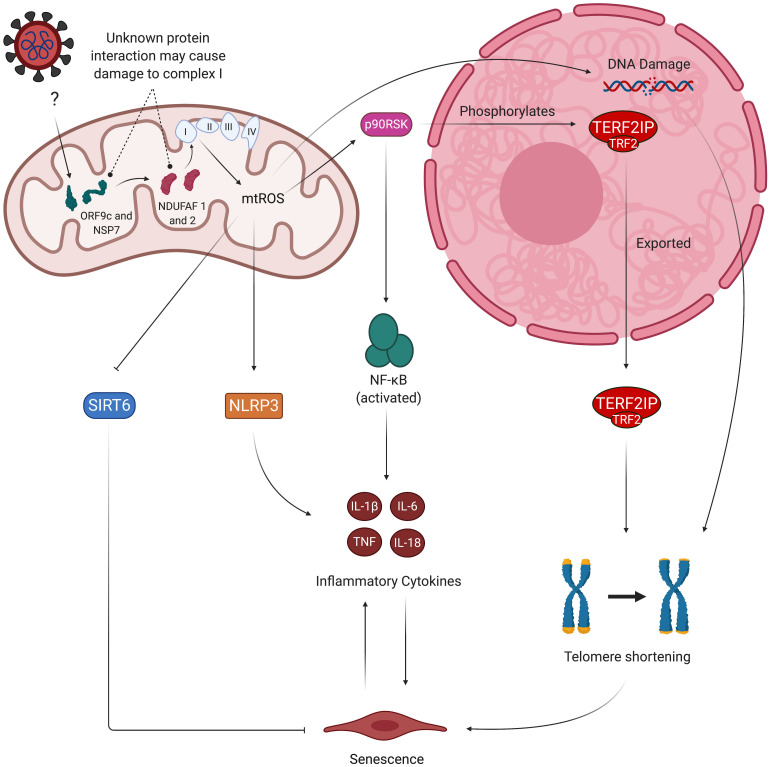
Viral hijacking of mitochondria may result in pro-senescent mitochondrial dysfunction and inflammation. Studies show evidence of viral proteins (ORF9c and NSP7) interacting with mitochondrial proteins (NDUFAF 1 and 2). While the exact nature of this interaction is unknown, NDUFAF 1 and 2’s roles in assembly of the ETC suggest the interaction may dysregulate proper formation of the ETC’s complexes, thus leading to increased mtROS production. mtROS-mediated activation of p90RSk then results in attenuation of anti-senescent SIRT6 and phosphorylation of TERF2IP, which is exported from the nucleus along with TRF2. This causes telomere shortening and subsequent induction of senescence and SASP. Other factors contributing to SASP include oxidative activation of NLRP3 inflammasome and NF-kB signaling mediated by the TERF2IP-TRF2 complex. Senescent ECs experience greater sensitivity to oxidative stress due to decreased activity of eNOS and secretion of inflammatory cytokines, thus perpetuating the cycle of oxidative stress and inflammatory SASP. mtROS, mitochondrial reactive oxygen species; eNOS, endothelial nitric oxide synthase; ORF9c, open reading frame 9c; NSP7, non-structural protein 7; p90RSK, 90 kDa ribosomal protein S6 kinase 1 (p90RSK); NF-kB, nuclear factor kappa beta; NLRP3, NLR family pyrin domain containing 3; TERF2IP, telomeric repeat-binding factor 2-interacting protein; TRF2, telomeric repeat-binding factor 2; SIRT6, sirtuin 6.

SARS-CoV-2 may also disrupt healthy mitochondrial function by directly hijacking the organelle. Wu et al., used a RNA-GPS program to predict that the SARS-CoV-2 RNA genome is strongly localized to the mitochondrial matrix, the production site of mtROS (Wu et al., 2020). The authors noted that hijacking the mitochondria may enable SARS-CoV-2 to produce double-membrane vesicles (DMVs) during viral replication – a mechanism used by other coronaviruses to conceal themselves from cellular defenses – which may explain the DMVs found in the lymph nodes of deceased COVID-19 patients (Bryce et al., 2020). These viral DMVs contain membrane-spanning complexes that are exposed to the cytosol and the DMV’s interior, and are thought to be the channel through which viral RNA is exported into the rest of the cell (Wolff et al., 2020). A separate study found that the SARS-CoV-2 proteins ORF9c and NSP7 interact with mitochondrial proteins NDUFAF 1 and 2, respectively (Gordon et al., 2020). NDUFAF 1 and 2 are of particular interest due to their role in assembly of the ETC complex I, which is one of the sources of mtROS (Mimaki et al., 2012). While the exact nature of this interaction is unknown, the prominence of COVID-19 phenotypes associated with excess mtROS production suggests that the ORF9c/NSP7/NDUFAF interaction may dysregulate assembly of complex I and subsequently increase mtROS levels. SARS-CoV-2 may also function similarly to its predecessor SARS-CoV, which suppresses the mitochondrial antiviral-signaling protein (MAVS) via its ORF-9b protein (Shi et al., 2014). It is also worth noting that some viruses can impair mitochondrial activity through several unexplored mechanisms; for example, the herpes simplex virus’s UL 12.5 protein depletes mitochondrial DNA through exonuclease activity, which can induce mitochondrial dysfunction and elevate mtROS production (Ide et al., 2001; Corcoran et al., 2009). Thus, direct viral and mitochondrial protein interactions may further contribute to mitochondrial dysfunction and its associated pro-senescent and pro-inflammatory phenotype in SARS-CoV-2-infected ECs (Figure 5). The mechanisms by which SARS-CoV-2 proteins localize to the mitochondria are currently unknown, and investigating such pathways may provide a target for preventing mitochondrial dysfunction in infected ECs (Singh et al., 2020). Potential consequences of SARS-CoV-2/mitochondrial protein interactions on mitochondrial dysfunction are summarized in the table below (Table 2).

**TABLE 2:** SARS-CoV-2 and mitochondrial protein interactions.

Viral proteins	Associated mitochondrial proteins	Role of mitochondrial proteins	Hypothesized interaction between viral/mitochondrial proteins	References
ORF9c, NSP7	NDUFAF1 and 2, NDUFB9	Involved in the assembly of Complex I in ETC	Dysfunctional/improper Complex I assembly, resulting in excess production of mtROS	Mimaki et al., 2012; Gordon et al., 2020
Catalytically dead NSP5	TRMT1	Performs dimethylguanosine base modification in both nuclear and mitochondrial tRNAs	Viral proteins localize TRMT1 exclusively to mitochondria, which increases ROS levels and sensitivity to oxidative stress	Dewe et al., 2017; Gordon et al., 2020
Membrane protein	ATP1B1, ATP6V1A, ACADM, AASS, PMPCB, PITRM1, PMPCA, COQ8B	Help assemble ATPase (ATP1B1 and ATP6V1A), acyl-CoA dehydrogenase (ACADM), mitochondrial processing peptidase subunit (PMPCB), and other metabolic components	Deregulation of normal mitochondrial metabolism	Gordon et al., 2020; Singh et al., 2020
ORF9b	TOMM70	Transports proteins into mitochondria and modulates anti-viral cellular defense pathways	Mutation or damage of TOMM70 is known to cause defects in oxidative phosphorylation (including elevated ROS production), and may also enable SARS-CoV-2 to suppress anti-viral response via the mitochondria	Shi et al., 2018; Gordon et al., 2020; Singh et al., 2020

## Sars-CoV-2 and EC Senescence

### Effects of mtROS Overproduction and Associated Senescence on ECs

Mitochondrial dysfunction and excess mtROS are associated with induction of premature EC senescence, which is characterized by shortened telomeres, arrest of cell growth, and a secretory phenotype in which the senescent cell releases various inflammatory cytokines and growth factors (Coppe et al., 2010; Vasileiou et al., 2019; Dominic et al., 2020a). With older adults experiencing significantly worse outcomes from COVID-19, examining the role of aging and accelerated senescence in SARS-CoV-2 is of great importance (Petersen et al., 2020). One study found that inhibiting complex I activity can induce a partial senescent EC phenotype, while interfering with complex III results in a much stronger senescent phenotype, supporting the idea that SARS-CoV-2 infection may initiate or exacerbate EC senescence by inducing ETC complex dysfunction (Moiseeva et al., 2009). NLRP3 inflammasome, which can be activated by excess mtROS from dysfunctional mitochondria, is associated with endothelial senescence and is thought to play a signaling role in promoting SASP in ECs (Yin et al., 2017). This most likely results from NLRP3’s increased production of IL-1β and subsequent time-dependent upregulation of p53, a transcription factor which is known to increase expression of target genes involved in cell cycle arrest and senescence (Yin et al., 2017).

Additionally, highly reactive hydroxyl radicals, which are formed via the previously discussed reaction between H_2_O_2_ and iron, cause telomeric DNA strand breaks by reacting with deoxyribose hydrogen atoms, which further exacerbates senescence and SASP (Mello Filho et al., 1984; Balasubramanian et al., 1998; Li et al., 2013; Coluzzi et al., 2019). mtROS-mediated activation of p90RSK also promotes senescence by phosphorylating telomeric repeat-binding factor 2–interacting protein (TERF2IP) and exporting the TERF2IP-TRF2 from the nucleus to the cytosol (Rovillain et al., 2011; Kotla et al., 2019). This cytosolic TERF2IP, which is phosphorylated at serine 205 by p90RSK, is responsible for activating NF-kB, a known contributor to premature aging and inflammation (Tilstra et al., 2011; Kotla et al., 2019). TERF2IP and TRF2 are also components of the Shelterin complex, which is responsible for preserving the structure of telomeres, so their depletion from the nucleus leads to telomere shortening and cellular senescence (Dominic et al., 2020a). The resistance of telomere damage to DNA repair mechanisms, such as non-homologous end joining, reinforces the idea that SARS-CoV-2-mediated increase in mtROS production has permanent pro-senescent and pro-inflammatory consequences on ECs (Fumagalli et al., 2012; Hewitt et al., 2012). Oxidative stress further contributes to EC senescence by reducing the expression of SIRT6 protein, which has anti-senescent and anti-inflammatory properties (Liu et al., 2014).

### Chronic Inflammatory Effects of Senescence

This proposed induction of senescence downstream of viral infection has additional negative implications for EC health. Previous research shows that senescent epithelial cells and fibroblasts are more susceptible to viral infection, as evidenced by higher levels of viral proteins from both influenza and *Varicella zoster* viruses in the senescent cells (Kim et al., 2016). Researchers hypothesize that senescence causes increased viral susceptibility by downregulating expression of interferons, a class of cytokines that are known to limit viral replication (Fritsch and Weichhart, 2016; Kim et al., 2016). Senescence may also impact the regulatory effects of mitochondrial ACE and ACE2, as aged rats exhibit higher mitochondrial ACE/ACE2 ratios that are associated with increased ROS production and oxidative stress (Escobales et al., 2019). Notably, the p53 transcription factor, which can initiate cell cycle arrest and senescence, can also reduce expression of manganese superoxide dismutase, thus perpetuating oxidative stress and subsequent senescence (Moiseeva et al., 2009; Passos et al., 2010). Thus, senescence engages in a vicious cycle of oxidative stress damage and inflammatory cytokine production, similar to the synergistic interactions between dysfunctional mitochondria and excess mtROS production (Figure 5).

This conclusion suggests that it is critical to prevent mitochondrial dysfunction and senescence following SARS-CoV-2 infection. Importantly, even after the inflammatory cytokine storm has passed and the patient has recovered from COVID-19, senescence remains irreversible, and senescent cells can accumulate long after their causative stress, as is the case in radiation-induced SASP (He and Sharpless, 2017). This is particularly relevant, as we have discussed how SARS-CoV-2 may induce continuous excess ROS production and p90RSK-mediated ERK5 S496 phosphorylation (Vu et al., 2018). Previous research indicates that HIV cART treatment increases long-term risk for cardiovascular disease by inducing p90RSK-mediated SASP, so SARS-CoV-2 infection may have a similar effect in inducing chronic senescence and inflammation in ECs (Singh et al., 2019). This further exacerbates the oxidative stress-mediated induction of SASP, production of inflammatory cytokines, and subsequent dysfunction in ECs.

Senescence also increases risk for EC apoptosis by decreasing phosphorylation and expression of endothelial NOS activity, thus reducing the protection NO offers against apoptosis and, in turn, chronically increasing risk of vascular leakage and lung injury (Hoffmann et al., 2001; Chambers et al., 2018). NO is known to deactivate caspase via S-nitrosylation of the caspase’s catalytic cysteine site, thus inhibiting one of the primary executors of apoptosis (Dimmeler and Zeiher, 1999; Liu and Stamler, 1999). Senescence-induced loss of NO exacerbates the already decreased levels of NO resulting from NO reacting with excess ROS and may explain the results of Varga et al.’s study, which found immunohistochemical staining patterns consistent with EC apoptosis in COVID-19 patients (Varga et al., 2020). Notably, extensive pulmonary EC apoptosis has been observed in the lungs of ARDS patients, further supporting the proposed role of senescence in promoting EC apoptosis and associated COVID-19 pathologies (Abadie et al., 2005). Together, these reports suggest that SARS-CoV-2 can induce SASP and subsequently promote chronic EC inflammation and further damage from oxidative stress.

### SASP-Produced TGF-β Perpetuates Redox Imbalance and Senescence

**FIGURE 6:**
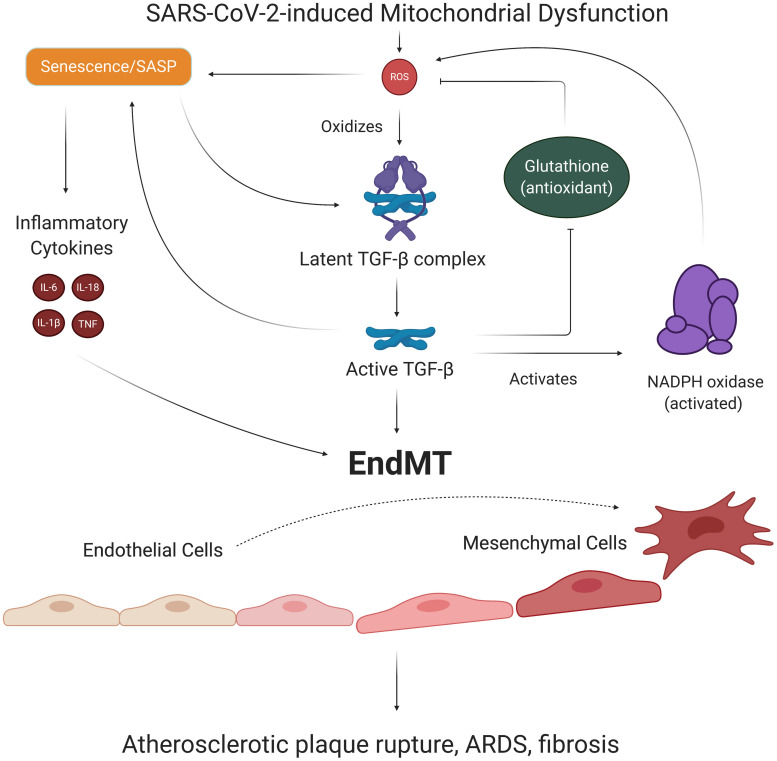
EndMT’s role in SARS-CoV-2-induced feedback loops. ECs with SARS-CoV-2-induced SASP secrete TGF-β, a growth factor known to promote the transition of ECs to mesenchymal, fibroblast-like cells. Oxidative stress is responsible for activating TGF-β, which activates Smad transcription factors that further promote senescence and induction of EndMT. The process of EndMT results in rupture-prone atherosclerotic plaques, weakening of the endothelial barrier, and cardiac and pulmonary fibrosis. Notably, TGF-β exacerbates redox imbalance and oxidative stress by increasing Nox subunit expression and suppressing production of the antioxidant glutathione. Thus, TGF-β and associated EndMT may be key players in our suggested long-term consequences of SARS-CoV-2 infection. EndMT, endothelial to mesenchymal transition; SASP, senescence-associated secretory phenotype; TGF-β, transforming growth factor beta.

One of the cytokines released in SASP is transforming growth factor beta (TGF-β), which has been viewed as a significant contributor to pulmonary pathologies in severe COVID-19 cases (Tominaga and Suzuki, 2019; Chen, 2020). While TGF-β is synthesized and secreted as an inactive complex bound to latency-associated protein, mtROS can oxidize the latency-associated protein and reduce its affinity for TGF-β, in turn freeing and activating TGF-β (Pociask et al., 2004; Liu and Desai, 2015). Once activated, TGF-β binds to TGF-β receptor 2, which recruits, phosphorylates, and activates TGF-β receptor 1 (Pardali et al., 2017). This TGF-β complex then phosphorylates various Smad proteins that translocate to the nucleus and bind to promoter regions in the DNA, upregulating and downregulating expression of various genes (Pardali et al., 2017). Smad proteins can repress expression of the telomerase reverse transcriptase gene, which results in reduced telomere length and subsequent senescence (Li et al., 2006; Tominaga and Suzuki, 2019). Smad proteins have also been shown to increase expression of the NADPH oxidase Nox 4 subunit, which produces ROS that fuel previously discussed pathways associated with mitochondrial dysfunction and increased mtROS production (Jiang et al., 2014a). TGF-β is thought to further induce redox imbalance by suppressing the expression of glutamate cysteine ligase, the rate-limiting enzyme in synthesis of the antioxidant glutathione, although the mechanism of this effect remains unclear (Liu and Gaston Pravia, 2010; Liu and Desai, 2015). These findings implicate TGF-β, mtROS, and SASP in a chronic feedback loop, wherein the production of TGF-β as a result of SARS-CoV-2-mediated SASP fuels further oxidative stress and inflammation (Figure 6).

### Potential Loss of EC Identity in SARS-CoV-2 Infection

Notably, TGF-β is one of the primary promoters of endothelial to mesenchymal cell transition (EndMT), a process by which ECs lose their endothelial characteristics and acquire mesenchymal, fibroblast-like phenotypes (Cho et al., 2018). Smad proteins activated by TGF-β complex interact with transcription factors and upregulate expression of pro-fibrotic genes that ultimately result in EndMT (Kovacic et al., 2019; Ma et al., 2020). Inflammatory cytokine signaling by TNFα and IL-6 can further promote EndMT by stimulating translocation of NF-kB to the nucleus, where NF-kB transcription factors bind to DNA and upregulate pro-EndMT genes (Mahler et al., 2013). Thus, the persistent state of oxidative stress and SASP in ECs may result in loss of EC identity downstream of SARS-CoV-2 infection (Figure 6). Investigating the mechanisms of these proposed SARS-CoV-2-induced pathways is critical because EndMT is a process associated with increased risk for atherosclerosis, pulmonary and cardiac fibrosis, and hypertension (Cho et al., 2018). In fact, one study found that about 35% of fibroblasts and mesenchymal cells in atherosclerotic plaques were of EndMT origin, and higher proportions of EndMT cells in plaques were associated with more rupture-prone plaque phenotypes (Evrard et al., 2016). Importantly, EndMT also results in elongation of ECs and loss of VE-cadherin complexes, phenomena which weaken the endothelial barrier and increase risk of pulmonary edema and injury (Ma et al., 2020). The potentially lethal damage EndMT can exert on the vasculature provides yet another key reason for investigating chronic feedback loops induced by SARS-CoV-2 infection.

## Clinical Reports and Observations of Sars-CoV-2 and EC Pathologies

The proposed feedback loops induced by SARS-CoV-2 infection may result in persistent oxidative stress and inflammation in ECs. This phenomenon may be largely implicated in various pathologies and complications that are commonly associated with hospitalized COVID-19 patients (Figure 1). The following sections will further discuss thrombosis, atherosclerosis, and ARDS as potential consequences of SARS-CoV-2-mediated changes in host redox status. The deleterious effects of oxidative stress in these conditions can be found in Table 3.

**TABLE 3:** Comprehensive overview of COVID-19-associated complications and pathologies.

Complication/pathology associated with COVID-19	Roles of oxidative stress and mitochondrial damage in relation to the pathology	Potential areas of study	References
Thrombosis	mtROS upregulates TF through PAR-1 and PAR-2; deletion of antioxidant murine thioredoxin reductase 2 induces prothrombotic phenotypes; crosstalk between mtROS-mediated inflammation and coagulation pathways	Role of mtROS in COVID-19-associated coagulopathy; TF expression amongst severely ill COVID-19 patients	Banfi et al., 2009; Foley and Conway, 2016; Kirsch et al., 2016
Atherosclerosis (AS) and Associated Cardiovascular Disease (CVD)	Increased production of oxLDL; induction of SASP in ECs; chronic endothelial inflammation and EC apoptosis; plaque progression and instability	The role of COVID-induced cytokine storm and p90RSK in the development and progression of AS; the role of inflammation in the vasculature as it relates to COVID-19-associated myocarditis	Madamanchi and Runge, 2007; Hulsmans et al., 2012; Hansson et al., 2015; Taleb, 2016; Yu and Bennett, 2016; Wolf and Ley, 2019
Acute Respiratory Distress Syndrome (ARDS)	mtROS and cytokines compromise barrier integrity; elevated plasma mtDNA and mtDNA DAMP levels	The mechanisms by which EC mitochondria may be damaged in COVID-19 induced ARDS	Simmons et al., 2017; Faust et al., 2020; Huang L. et al., 2020

### Thrombosis and Coagulopathy

#### Clinical Reports of Thrombosis and Coagulopathy in COVID-19 Patients

Many critically ill COVID-19 patients experience thrombotic complications as a result of inflammatory responses and EC damage in COVID-19-associated coagulopathy (CAC) (Al-Ani et al., 2020; Connors and Levy, 2020). An early Wuhan study of 183 patients with novel coronavirus pneumonia found that most patient deaths were significantly associated with increased levels of coagulation markers, including prothrombin time, activated partial thromboplastin time, fibrin degradation product, and dimerized plasma fragment D (D-dimer) (Tang et al., 2020). Several additional studies have found that elevated D-dimer levels are markedly associated with worse prognoses and outcomes for COVID-19 patients (Guan et al., 2020; Huang C. et al., 2020; Yang et al., 2020). In a comprehensive meta-analysis of several studies related to thrombosis and coagulopathy, Al-Ani et al., identified a venous thromboembolism frequency of 20% amongst 1765 patients, with severely ill ICU patients having significantly higher incidents of venous thromboembolism (Al-Ani et al., 2020). Moreover, the formation of microthrombi in SARS-CoV-2 infection differentiates COVID-19 from other respiratory illnesses, such as the flu, and may also lead to deleterious health outcomes, such as myocardial infarction and stroke (Ackermann et al., 2020; Magro et al., 2020). The high rate of thrombotic events in COVID-19 patients suggests that more clinical investigation is warranted, especially because D-dimer measurements may be non-specific predictors of severity and mortality in COVID-19 (Al-Ani et al., 2020; Liang et al., 2020). The following subsections will discuss the induction of CAC, as well as the potential long-term consequences of CAC in relation to oxidative stress.

#### Pathogenic Induction of COVID-19-Associated Coagulopathy

COVID-19-associated coagulopathy, like many other forms of coagulopathy, can be attributed to pathogenic interactions with the immune system. Proinflammatory pathways induced by SARS-CoV-2 infection can exacerbate the production of various pleiotropic cytokines that are known to maintain prothrombotic phenotypes in damaged ECs (Levi and van der Poll, 2010; Foley and Conway, 2016; Connors and Levy, 2020). Hence, the extensive cross-talk between inflammation and coagulation is likely responsible for acute and long-term thrombotic complications that are commonly seen in COVID-19 patients (Foley and Conway, 2016; Iba and Levy, 2018). In response to inflammatory stimuli downstream of SARS-CoV-2 infection, parenchymal cells, perivascular cells, and monocytes may release tissue factor (TF) into the bloodstream, allowing it to generate and form complexes with factor VIIa. These TF-VIIa complexes can then activate coagulation pathways that generate thrombin and continually recruit platelets to form blood clots (Drake et al., 1989; Fleck et al., 1990; Morrissey, 1995; Mackman et al., 2007; Foley and Conway, 2016). Additionally, TF-VIIa complexes trigger protease-activated receptor (PAR) signaling and interact with damage associated molecular patterns (DAMPs) that further damage endothelial cells through the upregulation of inflammatory processes, which releases more TF into the bloodstream (Bianchi, 2007; Demetz et al., 2010; Foley and Conway, 2016). Thus, the aforementioned cross-talk may create positive feedback loops that incur lasting tissue and endothelium damage, as well as accelerated coagulation.

#### The Role of Oxidative Stress in Coagulation and Thrombosis

Procoagulant activity induced by SARS-CoV-2 infection may partially be explained by oxidative stress resulting from excess mtROS production. Generally, ROS-mediated oxidative stress in ECs accelerates thrombotic processes through inflammatory stress (Loscalzo, 2003). More specifically, ROS originating from the mitochondria can mediate coagulation through PARs, a subset of G-protein coupled receptors that are associated with the upregulation of TF-VIIa coagulation when acted upon by thrombin and/or PAR selective agonist peptides (Riewald and Ruf, 2001; Banfi et al., 2009; Grimsey and Trejo, 2016). In human umbilical vein endothelial cells (HUVECs), mtROS, as opposed to other forms of ROS, have been shown to stimulate the production of TF through signaling pathways activated by PAR-1 and PAR-2 (Vouret-Craviari et al., 2003; Banfi et al., 2009; Liang et al., 2020). Another study found that mice exhibited increased fibrin deposition in the endothelium upon EC-selective deletion of murine thioredoxin reductase 2, a mitochondrial antioxidant enzyme that protects against overproduction of mtROS (Kirsch et al., 2016). In turn, elevated ROS originating from EC mitochondria contributed to prothrombotic phenotypes in arterial vessels (Kirsch et al., 2016). These evidences suggest that mtROS facilitates procoagulant activity in the vasculature, supporting the notion that mtROS upregulation can exacerbate and sustain prothrombotic EC phenotypes following SARS-CoV-2 infection (Figure 1). After patients have recovered from the acute phase of SARS-CoV-2 infection, persistent endothelial damage from chronic inflammation and oxidative stress may result in an imbalance of homeostatic coagulation that deviates toward a prothrombotic state. This potential shift in the coagulation balance is especially concerning in relation to the long-term thrombotic consequences of SARS-CoV-2 infection, as many hospitalized COVID-19 patients continue to develop deep vein thrombosis and pulmonary embolism, diseases that are known to display high probabilities of recurrence (Heit, 2012).

### Atherosclerosis and Associated Cardiovascular Disease

#### Atherosclerosis as a Risk Factor and Potential Consequence of COVID-19

Atherosclerosis (AS) is a chronic inflammatory disease process characterized by the buildup of plasma lipoproteins in sub-endothelial spaces. Damage to ECs and irregular blood flow patterns contribute to low-density lipoprotein (LDL) deposition and oxidation, which in turn promotes constant inflammation and EC injury in arterial walls (Libby, 2002; Gimbrone and Garcia-Cardena, 2016; Taleb, 2016; Zhu et al., 2018). Existing evidences suggest that AS is an independent risk factor in determining severity of COVID-19. Several studies have already identified how underlying CVD is associated with higher risk of SARS-CoV-2 infection and hospitalization (Cheng et al., 2020; Krittanawong et al., 2020; Mehra et al., 2020; Vinciguerra et al., 2020). Moreover, COVID-19 cases with worse prognoses and outcomes tend to be found in patient populations who are more likely to have established atherosclerosis, including but not limited to, the elderly and male populace (Libby, 2020). In support of these notions, Vinciguerra et al. (2020), have hypothesized that immune system dysregulation in pre-existing AS creates an ideal environment for SARS-CoV-2 entry into human cells. However, in addition to being a risk factor, AS may also be a long-term consequence of COVID-19, as SARS-CoV-2 infection may actively initiate chronic vascular endothelial dysfunction (Figure 1). The following subsections will discuss the ways in which SARS-CoV-2 may accelerate AS through increased oxidative stress and EC senescence.

#### Oxidative Stress in Atherosclerotic Processes

Atherosclerotic development is mediated by oxidative stress, as various forms of ROS modify LDL to form oxidized low-density lipoproteins (oxLDL) (Wolf and Ley, 2019). SARS-CoV-2 infection may further amplify ROS-mediated oxidation of LDL in atherosclerotic processes, representing a long-term and potentially lethal consequence of our proposed pathways on the vasculature. The binding of oxLDL to Toll-like receptors triggers signaling pathways through myeloid differentiation primary-response protein 88 and TIR domain-containing adaptor-inducing IFN-β, which activates macrophages and stimulates recruitment of proinflammatory cytokines and adhesion molecules to arterial walls (Edfeldt et al., 2002; Curtiss and Tobias, 2009; Falck-Hansen et al., 2013; van der Valk et al., 2016; Rhoads and Major, 2018). Furthermore, vascular uptake of oxLDL by receptors like LOX-1 can facilitate oxLDL-induced EC apoptosis (Li and Mehta, 2000, 2009). Increased conversion of LDL to oxLDL through SARS-CoV-2-mediated overproduction of ROS may serve to exacerbate these inflammatory and apoptotic processes in ECs, thereby instigating the development of AS in COVID-19 patients who have recovered from acute symptoms and complications.

oxLDL can also promote the production of excess mitochondrial O_2_^–^ through JNK activation, which downregulates Mn-SOD (Zmijewski et al., 2005; Takabe et al., 2010; Candas and Li, 2014). ox-LDL-mediated elevation of intracellular mtROS may be especially concerning in regard to COVID-19 because it can increase mtROS levels in the inflammatory feedback loops outlined by Figure 1. Overproduction of mtROS is strongly associated with damaged ECs in atherosclerotic lesions and is known to cause mitochondrial dysfunction and endothelial inflammation in AS (Madamanchi and Runge, 2007; Hulsmans et al., 2012; Li et al., 2013). Over time, sustained EC inflammation resulting from SARS-CoV-2-mediated oxidative stress would implicate further deposition and oxidation of LDL in subendothelial spaces, thereby reinforcing atherosclerotic development (Taleb, 2016). Furthermore, mtDNA damage is a potential downstream result of SARS-CoV-2 infection, and long-term buildup of damaged mtDNA directly promotes AS via additional ROS generation and EC apoptosis (Ballinger et al., 2002; Yu and Bennett, 2016). The aforementioned processes, which involve self-sustaining inflammation via cytokine induction and changes in redox status, also contribute to plaque destabilization and instability, which can result in cardiovascular complications like stroke and myocardial infarction (Mantovani et al., 2009; Hansson et al., 2015; Tabas et al., 2015; Fatkhullina et al., 2016; Zeng et al., 2018; Zhu et al., 2018). Thus, it is likely that SARS-CoV-2-induced feedback loops amplify AS and its associated CVDs.

#### Senescence in Atherosclerosis

Cellular senescence is known to play a large role in AS, as the development and rupturing of atherosclerotic plaques is related to premature vascular aging that promotes a chronic influx of leukocyte adhesion molecules and proinflammatory cytokines to vascular walls (Wang et al., 2007; Wang and Bennett, 2012; Donato et al., 2015). Several studies have found that shorter telomere length is a significant risk factor associated with the development of atherosclerotic CVDs (Chang and Harley, 1995; Samani et al., 2001; Brouilette et al., 2003, 2007; Wang et al., 2007). Minamino et al., found that inhibition of TRF2, a major stabilizing molecule of the telomeric Shelterin complex, induced senescence in human aortic endothelial cells, which promoted atherosclerosis (Minamino et al., 2002; Wang et al., 2007). As previously discussed, SARS-CoV-2 infection may also promote telomere shortening through the export of TERF2IP-TRF2 complex downstream of mtROS-mediated p90RSK activation (Figure 5). Thus, it is conceivable that oxidative stress initiated by SARS-CoV-2 infection could further contribute to senescent phenotypes that exacerbate AS development and progression in recovered COVID-19 patients.

#### Vascular Changes and CVDs: Chronic Consequences of SARS-CoV-2 Infection

Understanding the long-term effects of SARS-CoV-2 infection on the vasculature, particularly as it relates to AS and associated CVD, is of great importance, as most studies have only been able to evaluate the acute effects of COVID-19 during the infection period. Notably, some preliminary studies support the hypothesis that SARS-CoV-2 can inflict long-term damage on the vasculature. For example, a recent case report discusses the autopsy of a 31-year-old African American female who recovered from COVID-19 but passed away from other complications after initial discharge. Hematoxylin-eosin staining of the heart revealed vasculitis and endotheliitis in smaller cardiac vessels, which may be indicative of long-lasting injury to the vasculature of the heart via sustained inflammation initiated by SARS-CoV-2 infection (Fox et al., 2020). Additionally, the effects of COVID-19 on the vasculature may be related to sustained myocardial injury. Early cohort studies of COVID-19 patients identified myocardial injury as an acute manifestation of the COVID-19 disease process, with some of the studies citing elevated levels of troponins (Guo et al., 2020; Huang C. et al., 2020; Lippi et al., 2020; Shi et al., 2020; Yang et al., 2020). More recent evidence has found that myocardial injury can persist in patients who have recovered from viral infection. A cohort study of 100 German patients found that 60% of individuals presented with myocarditis after recovering from SARS-CoV-2 infection (Puntmann et al., 2020). Moreover, Lindner et al., noted that the myocardial tissue of recovered COVID-19 patients contains the SARS-CoV-2 viral genome, which partially supports hypotheses about direct viral myocardial injury to the heart through its highly expressed ACE2 receptors (Atri et al., 2020; Bansal, 2020; Lindner et al., 2020; Nicin et al., 2020). Although these studies particularly focus on inflammation of the cardiomyocytes, proinflammatory processes induced by viral infection in the cardiac vasculature largely mediate the pathogenesis of infectious myocarditis (Woudstra et al., 2018). Thus, chronic inflammation associated with ECs in the vasculature may partially explain the high incidence of myocarditis after acute recovery. Additional research is needed to verify the proposed role of chronic oxidative stress and inflammation in these long-term vascular changes associated with SARS-CoV-2 infection.

### ARDS and Lung Injury

#### Clinical Manifestations of ARDS

**FIGURE 7:**
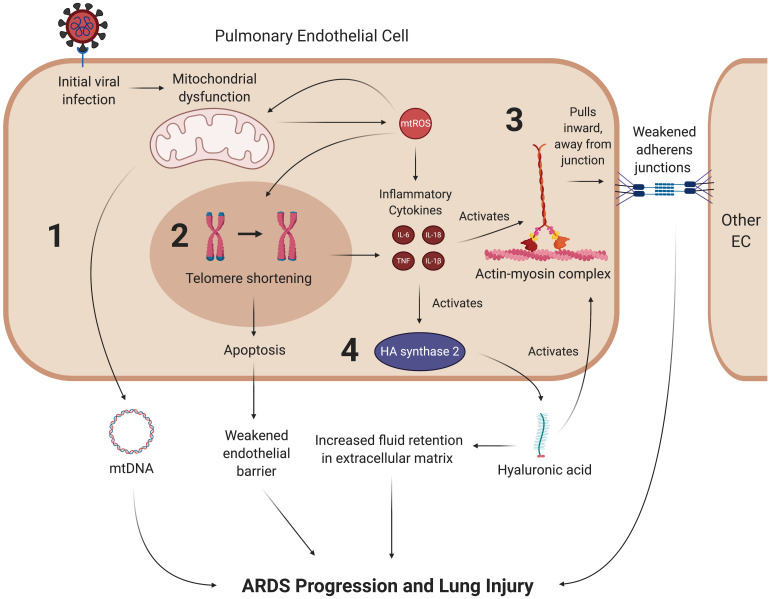
SARS-CoV-2 may promote lung injury and ARDS through chronic mitochondrial dysfunction and inflammation. Once the coronavirus initially induces mitochondrial dysfunction in ECs, it may create a feedback loop that continually promotes vascular fluid leakage and lung injury, with four mechanisms depicted here. (1) Dysfunctional mitochondria are thought to eject mtDNA into the plasma, which is associated with fluid leakage and ARDS. While it is hypothesized that mtDNA in the plasma contribute to ARDS progression, the exact mechanism of this pathway remains to be clarified. (2) mtROS synthesized by dysfunctional mitochondria can induce senescence through various pathways, most notably by reacting to form hydroxyl ions that damage telomeres. Senescent cells undergo apoptosis at a much higher rate than their healthy counterparts, which further weakens the endothelial barrier. (3) Inflammatory cytokines weaken the endothelial barrier by activating the contractile actin-myosin complex, which pulls the VE-cadherin inward and away from its binding partner, thus opening a gap in the endothelial barrier. (4) These cytokines also lead to the activation of hyaluronic acid synthase 2, which synthesizes hyaluronic acid. Hyaluronic acid is thought to promote ARDS by activating the actin-myosin complex through RhoA kinase pathways and increasing fluid retention in the lung’s extracellular space. mtROS, mitochondrial reactive oxygen species; IL, interleukin; TNF, tissue necrosis factor; mtDNA, mitochondrial deoxyribonucleic acid.

Acute respiratory distress syndrome (ARDS) is one of the primary clinical manifestations of COVID-19-induced respiratory failure. Several studies have acknowledged the rapid progression of ARDS as a comorbidity associated with high mortality rates amongst severely ill COVID-19 patients (Chen et al., 2020; Huang C. et al., 2020; Yang et al., 2020). Non-survivors of COVID-19 are much more likely to develop ARDS and require mechanical ventilation than those who eventually recover from the disease (Yang et al., 2020). Furthermore, one cohort study found that ARDS was the most common COVID-19 complication in its patient population, with over 90% of those ARDS patients requiring ICU care (Huang C. et al., 2020). The ARDS disease process commonly presents with dyspnea and hypoxemia as a result of widespread inflammation in the endothelial barrier of the lungs (Matthay and Zemans, 2011). Thus, damage to pulmonary microvascular endothelial cells (PMVECs) and their mitochondria are of particular importance in the discussion of COVID-19-induced ARDS and may also be implicated in long-term lung injury after patients have recovered from acute viral symptoms (Figure 7).

#### Interactions Between mtROS and the Pulmonary Endothelium Regulate ARDS Progression

The pulmonary endothelium regulates key homeostatic activities of the lungs through a wide variety of mechanisms, ranging from barrier integrity via inter-endothelial junctions to interactions with leukocytes and vasoactive mediators (Dudek and Garcia, 2001; Bazzoni and Dejana, 2004; Komarova and Malik, 2010; Sukriti et al., 2014; Millar et al., 2016). Under optimal conditions, PMVECs typically promote anti-inflammatory pathways. However, in ARDS, the disruption of these mechanisms by cytokines and other effector molecules reconfigure, or “activate”, the ECs, causing them to display proinflammatory phenotypes (Zimmerman et al., 1999; Millar et al., 2016; Gonzales and Verin, 2018). In particular, mtROS and inflammatory cytokines compromise barrier integrity by stimulating the actin-myosin contractile apparatus. The activation of this apparatus causes contraction of stress fibers that pull pulmonary ECs away from each other and weaken their corresponding intracellular junctions, leading to increased pulmonary edema that is characteristic of ARDS (Hurley, 1982; Dudek and Garcia, 2001; Millar et al., 2016). More specifically, the compromised barrier integrity results from tensile damage to the VE-cadherin-catenin complexes in adherens junctions (Komarova and Malik, 2010; Kasa et al., 2015). It is also worth noting that paracellular gap formation stimulated by EC activation in ARDS increases pulmonary capillary permeability, which also contributes to fluid leakage and inflammation (Gonzales et al., 2015; Palumbo et al., 2017). Furthermore, the inflammatory cytokines TNF and IL-1β activate glucuronidases, which subsequently activate hyaluronic acid synthase 2 (Teuwen et al., 2020). This results in increased production of hyaluronic acid in the extracellular matrix, a process that is hypothesized to further upregulate endothelial permeability and accumulation of fluid in the extracellular space; indeed, higher levels of hyaluronic acid have been correlated with more severe lung injury in ARDS patients (Esposito et al., 2017; Lonati et al., 2018). Hyaluronic acid binding protein induces PAR signaling that results in activation of RhoA, a GTPase that promotes actin stress fiber formation and actin-myosin contraction, which further weakens the junctions of the endothelial barrier (Mirzapoiazova et al., 2015; Pronk et al., 2019). All of the aforementioned mtROS-mediated pathways suggest a potential means by which oxidative stress downstream of SARS-CoV-2 infection can induce ARDS and long-term lung injury (Figure 7).

#### The Link Between ARDS and Plasma Mitochondrial DNA

Acute respiratory distress syndrome pathology may also be characterized by mitochondrial dysfunction as a response to inflammatory events in ECs. Simmons et al., investigated the role of mtDNA DAMPs as a response to inflammatory stimuli and found that serum concentrations of mtDNA DAMPs were significantly higher in in patients who developed ARDS (Simmons et al., 2017). Although their data does not necessarily show which mt-derived DAMP is associated with ARDS, it may suggest that mtDNA is being ejected into the extracellular environment as a result of mitochondrial damage (Simmons et al., 2017). In support of these data, a cohort study conducted by Faust et al., found that trauma- and sepsis-induced ARDS were significantly associated with elevated plasma mtDNA levels (Faust et al., 2020). Furthermore, emerging evidence suggests that higher plasma mtDNA levels are positively associated with an increased risk of death in ARDS patients (Huang L. et al., 2020). Collectively, these findings suggest that EC mitochondrial dysfunction is a key mediator of ARDS progression. The deleterious roles of mtROS and EC mitochondria in previous studies of ARDS pathology suggest that SARS-CoV-2-mediated oxidative stress may cause downstream lung injury through similar mechanisms (Figure 7).

## Potential Therapeutics

**FIGURE 8:**
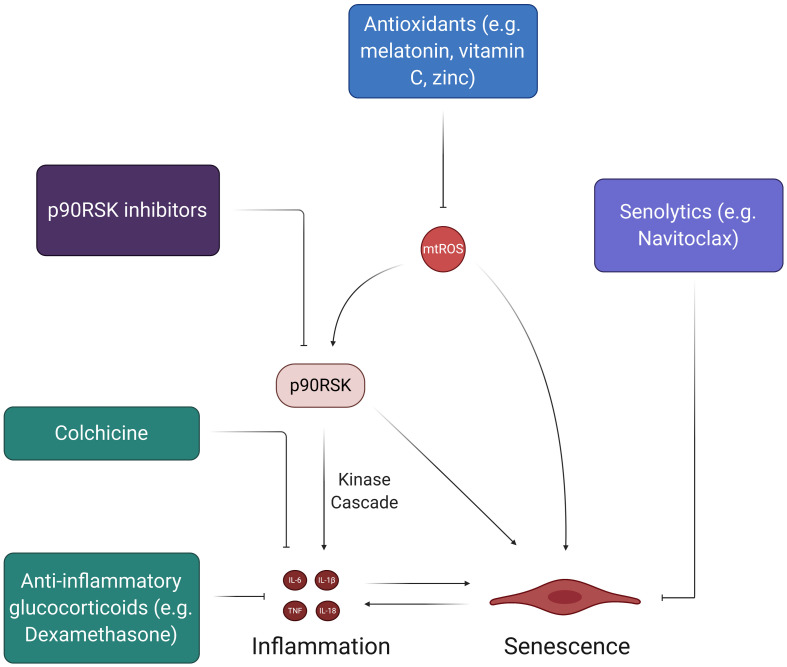
Therapies to address SARS-CoV-2-induced feedback loops in ECs. While there have been many therapies proposed to halt the spread of the coronavirus pandemic, the ones depicted here address the feedback loops involving oxidative stress and inflammation. The chronic, self-feeding nature of mtROS, inflammation, and senescence provide various targets for treatment. For example, antioxidants can prevent the accumulation of excess mtROS, thus reducing the inflammatory effects of mtROS-induced cytokine production and SASP. Likewise, senolytics, which induce apoptosis of senescent cells, can reduce the pro-inflammatory state associated with SASP and prevent further induction of senescence in the endothelium.

Ongoing clinical investigation is focused on finding novel therapeutics for treating COVID-19, as very few drugs have proven to be efficacious in improving outcomes for critically ill patients. Although the antiviral drug Remdesivir has displayed improved recovery times in some studies, it does not conclusively improve mortality rates amongst COVID-19 patients and has been shown to provide minimal clinical benefits in other studies (Beigel et al., 2020; Grein et al., 2020; Wang Y. et al., 2020). Based on the limited scope of current antiviral treatment options, further investigation into new therapeutic targets is warranted. The signaling mechanisms related to the aforementioned COVID-19 complications and pathologies may serve as potential targets for novel therapeutics. Figure 8 discusses potential therapeutics that may provide acute and long-term benefits by alleviating inflammation, oxidative stress, and senescence.

### Anti-inflammatory Therapies

The cytokine storm in COVID-19 plays a significant role in initiating potent and widespread inflammatory EC damage. Thus, much of the current work concerning acute treatment of the cytokine storm via anti-inflammatory drugs can help to mitigate the initial damage that may lead to long-term dysfunctional phenotypes in multisystem ECs. Two of the most discussed drugs in recent literature and popular media are chloroquine and hydroxychloroquine, which are meant to treat SARS-CoV-2 infection by preventing the induction of inflammatory cytokines like TNF and IL-6 (Jean et al., 2020; Schrezenmeier and Dorner, 2020; Ye et al., 2020). Although these drugs were shown to have some positive effects in preliminary cohort studies, newer studies suggest that chloroquine and hydroxychloroquine do not significantly correlate to recovery and provide little to no benefits as treatment options (Arshad et al., 2020; Boulware et al., 2020; Gao et al., 2020; Gautret et al., 2020; Geleris et al., 2020; Mahevas et al., 2020; Mitja et al., 2020; Rosenberg et al., 2020). A 19-year-old female taken off of HCQ found better results and full recovery when given colchicine, an anti-inflammatory inhibitor of NLRP3 and IL-6 that is normally implicated in the treatment of CVDs (Leung et al., 2015; Kajikawa et al., 2019; Tardif et al., 2019; Recalcati et al., 2020). The GRECCO randomized clinical trial (RCT) tested colchicine as a potential therapeutic for COVID-19 and found that it significantly reduced clinical deterioration in treatment groups relative to control groups (Deftereos et al., 2020). Interestingly, colchicine also displayed anti-thrombotic properties in COVID-19, as treatment groups in the study exhibited significantly lower D-dimer levels (Deftereos et al., 2020; Rabbani et al., 2020). More importantly, however, the recent RECOVERY trial found that Dexamethasone, an anti-inflammatory glucocorticoid, significantly reduced mortality in COVID-19 patients who needed respiratory support (RECOVERY Collaborative Group et al., 2020). Although these results are promising, more clinical studies need to be done to confirm the efficacy of Dexamethasone so that it can potentially become a widely used therapeutic. The RECOVERY trial presents an interesting and novel proposal for using anti-inflammatory glucocorticoids in COVID-19 treatment (RECOVERY Collaborative Group et al., 2020). Future studies should evaluate the safety and efficacy of Dexamethasone and other glucocorticoids as a means of mitigating the downstream effects of the initial cytokine storm that may propagate acute and chronic inflammation.

### The Prospect of Confronting EC Senescence

The selective targeting of senescence may be another means by which the potential acute and long-term effects of SARS-CoV-2 infection can be mitigated, as stimulated senescent cells produce SASP proteins that can facilitate systemic inflammation. Based on recent evidences, some of the most promising anti-senescent therapeutics include treatments that decrease SASP biomarker levels (McHugh and Gil, 2018; Amaya-Montoya et al., 2020). Most studies that have displayed significant inhibition of SASP biomarkers have focused on the suppression of signaling mechanisms (e.g., NF-kB, mTOR, p38MAPK, BRD4) in fibroblasts rather than ECs (Chien et al., 2011; Freund et al., 2011; Moiseeva et al., 2013; Laberge et al., 2015; Tasdemir et al., 2016; Wang et al., 2017). However, inhibition of the pro-inflammatory JAK-STAT pathway and TNF-α decreases SASP expression in HUVECs (Xu et al., 2015; Prattichizzo et al., 2016). The central idea of reducing SASP biomarker levels through the inhibition of signaling pathways also suggests the potential of blocking p90RSK activity, which activates and maintains senescent EC phenotypes through NF-kB activation and telomere shortening (Figure 5). Future studies should explore the use of p90RSK inhibitors, such as FMK-MEA, as potential therapeutics for EC senescence caused by SARS-CoV-2 infection. Furthermore, senescent cells can be effectively targeted through a class of drugs known as senolytics, which are aimed at inducing apoptotic pathways in senescent cells (McHugh and Gil, 2018; Amaya-Montoya et al., 2020). For example, Navitoclax, a potent senolytic drug, works by impeding the actions of anti-apoptotic Bcl proteins in senescent cells and has robust apoptotic outcomes in HUVECS (Zhu et al., 2016; McHugh and Gil, 2018). Although more investigation into the efficacy of senolytic drugs is needed, they may help in preventing acute and chronic complications of COVID-19 by reducing senescent cell burden in recovering patients.

### Antioxidant Therapies

Antioxidant therapies are another class of potential therapeutics for COVID-19 and its associated pathologies. Theoretically, antioxidants would attenuate the extensive presence of mtROS within ECs and could prevent the chronic oxidative damage proposed by this review (Figure 8). This line of thinking explains the proposition of using supplementary antioxidant substances like vitamin C and melatonin in future COVID-19 treatment-related studies (Carr, 2020; Zhang et al., 2020). In particular, vitamin C has been linked to improved outcomes in septic patients, making it a conceivable candidate for study in hospitalized COVID-19 patients (Fowler et al., 2014; Zabet et al., 2016; Marik et al., 2017). However, although many licensed antioxidants are meant to target COVID-19 related pathologies (i.e., inflammation, atherosclerosis, CVDs), studies on the safety and/or efficacy of these types of treatment options in humans are limited (Zhang et al., 2016). Moving forward, more cohort studies on antioxidant treatments are necessary, as many practitioners continue to treat COVID-19 patients with antioxidants, such as IV vitamin C and zinc, without extensive support from clinical data.

### Vaccination Strategies

Several collaborative efforts enabled the development of variety of vaccine candidates to prevent the spread and to provide the public with protection and immunity against the virus (Dong et al., 2020). Notably, the phase three clinical trial results from vaccine candidates from Pfizer and Moderna seem to be tremendously successful, with success rates of 95 and 94.1%, respectively (NIH, 2020a, b). Other notable vaccine candidates in early and late clinical trials may serve as an alternative and might help mitigate the cold chain problem (Dong et al., 2020). The long-term efficacy and safety of the vaccines are still being tested; specifically the effect of vaccines on ECs and vasculature are still unknown. Although vaccines are the most critical and effective measure to stop the spread and elimination of SARS-CoV-2, the long-term oxidative stress and inflammatory effects of the virus on ECs, pericytes and vasculature in recovering patients necessitates the importance of investigating the above-mentioned therapeutic strategies.

## Conclusion and Future Directions

As the pandemic continues to rage worldwide, management of COVID-19 is a top public health priority for the global community. Some of the most significant COVID-19 complications, such as thrombosis, CVDs, and ARDS, are direct results of inflammation and mitochondrial oxidative stress in ECs. Through Ang II-mediated NADPH oxidase activation, viral/mitochondrial protein interactions, and dysregulation of mitochondrial metabolism, SARS-CoV-2 infection may induce mitochondrial dysfunction that leads to excess production of mtROS. The virus may further induce mitochondrial dysfunction through direct hijacking of the mitochondria by viral proteins and dysregulation of mitochondrial metabolism. By activating pathways such as NF-kB and the NRLP3 inflammasome, the elevated levels of mtROS can induce chronic inflammation and senescence in ECs, thus contributing to the pathologies observed in severe COVID-19 cases. Critically, oxidative stress and EC inflammation are involved in feedback loops that would pose a highly increased risk for dysfunctional endothelial pathologies even after the patient has recovered from the virus. Therefore, we suggest SARS-CoV-2 should not be viewed just as an acute inflammatory syndrome, but also as an instigator of long-term oxidative stress, inflammation leading to endothelial and vascular dysfunction. Although the recent clinical trials of COVID-19 vaccines bodes well for preventing future infections, the number of recovering and infected patients continues to rise worldwide, necessitating investigation of the virus’s long-term deleterious effects. While significant research is being done to develop vaccinations and acute treatment options, it is just as important to investigate the potential long-term damage done to the endothelium by the virus. To prevent a future outbreak of pulmonary and cardiovascular mortalities among recovered COVID-19 patients, it is essential to investigate SARS-CoV-2’s potential role in chronic oxidative stress and inflammation in ECs. Future research should also evaluate changes in EC redox status after COVID-19 recovery, potentially through analysis of oxidative stress biomarkers and redox-sensitive proteins like p90RSK and NLRP3. Researchers should also conduct clinical trials to verify the efficacy of antioxidant, anti-inflammatory, and senolytic therapeutics that may attenuate the proposed long-term effects on ECs. AS and CVD are important consequences of several systemic inflammatory syndromes (Fong, 2009; Teixeira and Tam, 2017). Interesting observations of SARS-CoV-2’s interactions with ECs will uncover previously unknown mechanisms of ECs and their role in pathogenesis of inflammatory syndromes observed in other diseases. These understandings will be crucial to mitigate and treat several infectious diseases of the present and as we step into the uncharted post-COVID era.

## Author Contributions

RC and AM prepared the manuscript and figures and collected sources. AD designed and ideated the article, collected sources, prepared and revised the manuscript and figures. N-TL provided guidance, critical feedback and supervised the entire project. All authors contributed to the article and approved the submitted version.

## Conflict of Interest

The authors declare that the research was conducted in the absence of any commercial or financial relationships that could be construed as a potential conflict of interest.

## Glossary

COVID-19, Coronavirus disease 2019; SARS-CoV-2, severe acute respiratory syndrome coronavirus 2; EC, endothelial cell; ACE2, angiotensin-converting enzyme 2; Ang II, angiotensin II; AT_1_R, angiotensin II type 1 receptor; MAS1, MAS1 proto-oncogene; TMPRSS2, transmembrane protease, serine 2; ADAM17, ADAM metallopeptidase domain 17; mtROS, mitochondrial reactive oxygen species; ETC, electron transport chain; SOD, superoxide dismutase; MMP, mitochondrial membrane potential; mitoK_ATP_, mitochondrial ATP-sensitive potassium channel; Nox, nicotinamide adenine dinucleotide phosphate oxidase; Rac1, Ras-related C3 botulinum toxin substrate 1; IL, interleukin; TNF, tissue necrosis factor; ERK5, extracellular-signal-regulated kinase 5; p90RSK, p90 ribosomal S6 kinase; PKC, protein kinase C; PARP-1, Poly ADP-ribose polymerase-1; JNK, c-Jun N-terminal kinases; TCA, tricarboxylic acid; SIRT1, sirtuin 1; PGC-1α, peroxisome proliferator-activated receptor gamma coactivator 1-alpha; DMV, double-membrane vesicle; NLRP3, NLR family pyrin domain containing 3; NF-kB, nuclear factor kappa beta; SASP, senescence-associated secretory phenotype; TERF2IP, telomeric repeat-binding factor 2–interacting protein; TRF2, telomeric repeat-binding factor 2; TGF-β, transforming growth factor beta; EndMT, endothelial to mesenchymal cell transition; TF, tissue factor; PAR, protease-activated receptor; oxLDL, oxidized low-density lipoprotein; ARDS, acute respiratory distress syndrome; PMVEC, pulmonary microvascular endothelial cell; DAMPs, DNA damage-associated molecular patterns; CAC, COVID-19-associated coagulopathy; AS, atherosclerosis.
